# Virtual cardiac monolayers for electrical wave propagation

**DOI:** 10.1038/s41598-017-07653-3

**Published:** 2017-08-11

**Authors:** Nina Kudryashova, Valeriya Tsvelaya, Konstantin Agladze, Alexander Panfilov

**Affiliations:** 1 Department of Physics and Astronomy, Gent University, Gent, 9000 Belgium; 2 Laboratory of Biophysics of Excitable Systems, Moscow Institute of Physics and Technology, Dolgoprudny, 141701 Moscow Region Russia

## Abstract

The complex structure of cardiac tissue is considered to be one of the main determinants of an arrhythmogenic substrate. This study is aimed at developing the first mathematical model to describe the formation of cardiac tissue, using a joint *in silico*–*in vitro* approach. First, we performed experiments under various conditions to carefully characterise the morphology of cardiac tissue in a culture of neonatal rat ventricular cells. We considered two cell types, namely, cardiomyocytes and fibroblasts. Next, we proposed a mathematical model, based on the Glazier-Graner-Hogeweg model, which is widely used in tissue growth studies. The resultant tissue morphology was coupled to the detailed electrophysiological Korhonen-Majumder model for neonatal rat ventricular cardiomyocytes, in order to study wave propagation. The simulated waves had the same anisotropy ratio and wavefront complexity as those in the experiment. Thus, we conclude that our approach allows us to reproduce the morphological and physiological properties of cardiac tissue.

## Introduction

Electrical waves of excitation propagate through the heart and initiate cardiac contraction. Abnormalities in wave propagation may result in cardiac arrhythmia. According to a report published by the World Health Organisation^1^, cardiovascular diseases account for the highest number of deaths in the world, among which, around 40% occur suddenly and are caused by arrhythmias. Thus, understanding the principle of wave propagation is essential for decreasing cardiovascular mortality.

The electromechanical function of the heart is performed by excitable cells called cardiomyocytes (CMs), which are capable of generating an action potential and of mechanical contraction. In addition to CMs, cardiac tissue also contains other cells, the most abundant of these being fibroblasts (FBs). FBs are small inexcitable cells present in the heart in large numbers. Excess fibrous tissue, or fibrosis, can substantially affect wave propagation. In addition to FBs, there exist structural extracellular proteins (e.g. collagens), which form the extracellular matrix (ECM) and affect the CM phenotype^2^. The latter is essential for proper mechanical functioning of the heart^3^ and for uninterrupted electrical signal propagation^4^. The interaction between CMs, FBs, and extracellular proteins results in the formation of a complex tissue texture. Such a texture changes substantially during most cardiac diseases, via a process called *remodelling*. Cardiac remodelling is considered to be one of the major determinants of arrhythmogenicity in cardiac tissue^5, 6^. However, gradual changes in tissue architecture that lead to remodelling are hidden from observation, and there exists no direct method to study it in patients^7^. Thus, an alternative approach to understand the principles of formation of normal and abnormal cardiac tissue, and the possibility to predict their changes during remodelling are of great interest.

The most logical way to approach this problem, is to represent knowledge about such processes in terms of a mathematical model of structural tissue formation. This model should be based on extensive experimental data, which can be used to explain the observed textures and to develop methods to control remodelling. Ideas related to the importance of such a model have been widely discussed in strategic papers on cardiac computer modelling^8, 9^. However, this approach has not yet been realised. On the other hand, tissue growth models are extensively used in developmental biology. One of the most advanced approaches in this field is the large Potts model approach, or, in particular, the Glazier-Graner-Hogeweg (GGH) model^10–12^. Various processes of cell- and tissue morphogenesis, e.g, the process of root growth^13^, angiogenesis^14^, stem cell differentiation^15^, morphogenesis of *Dictyostelium discoideum* ^16^, epidermal formation^17^ and vascular system development^18^, etc. have been described using this model.

The aim of this study is to introduce GGH models to the field of cardiac research and to develop a detailed tissue formation model for cardiac tissue. We develop this model for a classical experimental model system–cardiac cell culture^19^. Such cultures are widely used in stem cell-^20–22^ and regenerative medicine research, as they can be expected to reproduce architectural properties close to those of real cardiac tissue. In addition, cell cultures provide a valuable tool to study the mechanisms of cardiac arrhythmias, especially rotors^23^. We are mainly interested in cells cultured on nanofibrous substrates that resemble the ECM of the heart. If these nanofibres are aligned, the tissue obtains structural and functional anisotropy^24^, which is one of the main factors affecting wave propagation in the heart. This nanofibre-based experimental system is ideal for our purpose, because here, the electrical properties of the cardiac cultures are closely related to their morphology. Such a system can be directly monitored with optical recordings^23^ that facilitate validation of the model at each step of its development. Therefore, we have developed GGH models for this particular experimental model.

Our paper is organised as follows. In the first section of the Results, we describe the experimental model for cardiac tissue formation and specify the cell shape acquisition procedure. In the second section, we focus on the mathematical GGH-type model for cardiac tissue, provide validated coefficients for the model and demonstrate its capabilities. In the third section, we demonstrate wave propagation patterns in isotropic and anisotropic samples. Next, we discuss our results in relation to other cell-based discrete models and available experimental data, and possible future work in this direction. Finally, we provide a detailed description of the methods and algorithms used in our study.

Taken together, we show that GGH models can quantitatively reproduce cardiac cell shapes, explain their elongation along a fibrous substrate and reproduce the experimental data. We conclude that it is a valuable tool for studying the connection between the morphology and function of cardiac tissue.

## Results

### Experimental study of cell shapes in cardiac tissue

#### Simplified cases of cardiac morphogenesis

In computational studies, we reproduced the experimental setting of neonatal rat cells cultured on a polymer nanofibrous scaffold. Cell cultures grown on such an artificial ECM, which imitates the ECM of the heart, effectively reproduce the anisotropy of cardiac tissue^24^. This engineered cardiac tissue has a complex structure resulting from cell–cell and cell–substrate interactions. During model development, we also considered simpler experimental situations that prevented cell–cell or cell–nanofibre interaction. To achieve this experimentally, we seeded the cells at a low density so that they could not touch one another. We also used a uniform scaffold without fibres, in which the cells spread equally in all directions.

**Figure 1:**
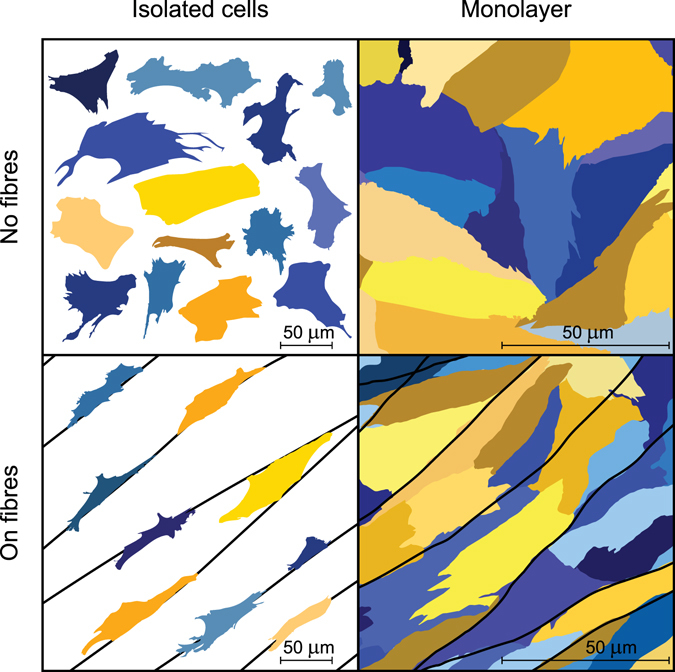
Experimental cases considered in our study. The first column shows isolated cells seeded at a low density to avoid cell–cell interaction. The second column represents cells in the monolayer. The substrate was isotropic in the upper row, whereas in the second row, nanofibres were added. Cardiomyocytes are shown in yellow tints, whereas fibroblasts are shown in blue tints. This image was based on immunohistochemical data but was refined for illustrative purpose.

As a result, we reproduced in an experiment, the following four different conditions: non-interacting cells with and without polymer nanofibres and monolayers with and without polymer nanofibres (see Fig. 1). Here, CMs and FBs are shown in different shades of yellow and blue, respectively. Finally, we developed a procedure for cell shape analysis, which we used to validate our mathematical model.

#### Identification of cell shapes in the experiment

**Figure 2:**
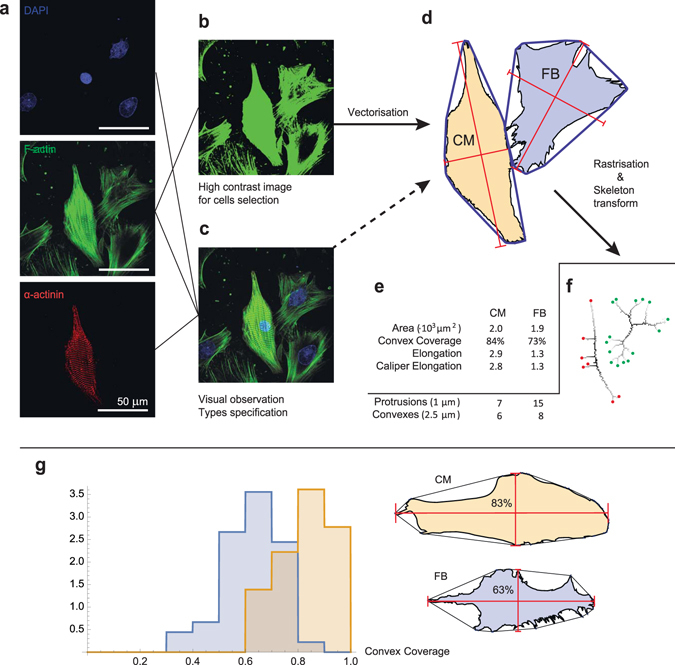
Algorithm for cell shape analysis in the experiments. Cardiac cells were observed experimentally with the use of immunohistochemical techniques. Panel (a) shows raw data on cells stained with DAPI (DNA, blue), phalloidin (F-actin, green) and monoclonal anti-*α*-actinin antibody (*α*-actinin, red). Sub-figures (**b**) and (**c**) show the processed data used for cell contour selection and cell type classification. In (**b**), the contrast of F-actin staining was enhanced for cell contour selection. In (**c**), three channels were merged to obtain better cell representation for classification (cardiomyocyte vs. fibroblast). Sub-figure (**d**) shows the cell images obtained after segmentation. The convex hull is shown as a blue line around each cell. These images were used to obtain parameters such as area, convex hull coverage and elongations. Cell images were then rasterised with two resolutions (1 *μm* and 2.5 *μm*), and skeleton transform was applied (**f**). The number of skeleton endpoints at a high resolution was considered as the number of protrusions. All the measured parameters are listed in (**e**). For details, see section III B. (**g**) Distribution of the convex coverage for two cell types. CM–cardiomyocyte (n = 36), FB–fibroblast (n = 45). The histogram shows that FBs cover a much smaller area within the convex hull than CMs. The cell shapes of the median samples in the experimental database are shown on the right.

We stained cell cultures with DAPI (DNA, blue), phalloidin (F-actin, green) and monoclonal anti-*α*-actinin antibody (*α*-actinin, red). From the *α*-actinin image, we were able to discriminate CMs from FBs (see Fig. 2). We used F-actin staining images for cell shape acquisition, and DAPI, for cell counting. Details of the cell shape acquisition procedure and further analysis can be found in Section III B. After the procedure, we collected the database of cell shapes for each of the four conditions that we studied.

We developed a custom code (in Wolfram Mathematica) for cell shape analysis (for details, see the Methods section III B). Using this code, we tested many standard morphological parameters and found the most valuable ones for the validation of the mathematical model. These parameters are cell spreading area, elongation, convex coverage, real number of focal adhesions (“protrusions”) and number of pronounced convexes of the cell periphery (“convexes”) for 2.5 *μ*m resolution (for details, see Section III B).

Using this approach, we collected the data from 103 isolated cells on a uniform substrate, 77 isolated cells on fibres, 127 cells in the isotropic monolayer and 294 cells in monolayers on fibres.

Most of the cells were classified as CMs or FBs according to the level of *α*-actinin expression and actin cytoskeleton development. Some cells with controversial characteristics (for example, *α*-actinin positive, but without developed cytoskeleton) were rejected. All measured parameters and statistics for the classified cells are presented in Table 1. These parameters serve as an important dataset to verify and tune our model.

**Table 1:** Cell shape characteristics measured in the experiment.

	Isolated cells		Monolayer		
	CM	FB	CM	FB	
	*n* = 36	*n* = 45	*n* = 67	*n* = 53	
Area(·10^3^ *μm* ^2^)	2.5 ± 1.0	2.1 ± 1.0	1.0 ± 0.4	0.9 ± 0.3	No fibres
Convex Coverage	84 ± 9%	66 ± 11%	79 ± 11%	70 ± 10%	
Elongation	1.7 ± 0.8	1.7 ± 0.6	2.1 ± 0.8	2.0 ± 0.8	
Protrusions (1 *μm*)	13.1±5.8	16.1 ± 5.7	7.6 ± 3.2	8.2 ± 3.3	
Convexes (2.5 *μm*)	5.3±1.5	6.4 ± 2.4	3.3 ± 1.3	4.0 ± 1.3	
	*n* = 32	*n* = 40	*n* = 34	*n* = 27	On fibres
Area(·10^3^ *μm* ^2^)	1.4 ± 0.8	1.3 ± 0.8	0.6 ± 0.2	0.6 ± 0.2	
Convex Coverage	80 ± 9%	63 ± 12%	81 ± 9%	60 ± 13%	
Elongation	3.0 ± 1.4	2.2 ± 1.4	3.2 ± 0.9	2.6 ± 1.0	
Protrusions (1 *μm*)	6.8 ± 3.9	12.6 ± 6.0	4.6 ± 2.5	5.8 ± 2.3	
Convexes (2.5 *μm*)	2.8 ± 1.6	4.8 ± 2.5	2.4 ± 1.2	3.1 ± 1.6	

One interesting observation is that some CMs can be distinguished from FBs, on the sole basis of cell shape, even in the absence of staining. The most important parameter for types specification is the convex hull coverage, which is the ratio of cell spreading area to the area of its convex hull. CMs normally occupy around 80% of a convex hull, whereas FBs cover only 60%–70%, and have much deeper concaves. Figure 2 shows the distributions for the convex coverage of CMs and FBs. A substantial difference exists between the convex coverage of these two types of cells. Statistically, CMs and FBs have a considerably different median convex coverage (*p*-value <10^−3^, *n*_CMs_ = 36, *n*_FBs_ = 45) (see Fig. 2g). No CMs with convex coverage lower than 60%, and no FBs with convex coverage higher than 90% were observed in experiment. Therefore, for 40% of FBs and for 30% of CMs, the cell type could be determined, relying only on the cell shape.

It is well known, that FBs have 15–30 times smaller volume than CMs^25, 26^. However, in cardiac monolayers (shown in Fig. 1) FBs occupy almost the same area as CMs. In our cell cultures, the height of the cells measured with the confocal microscope was approximately 1 *μ*m for the FBs (everywhere, except for the nuclei), and 7 *μ*m for the CMs. Therefore, the volumes of the cells in two-dimensional cell cultures are still similar to those in three dimensions. The resulting spreading area is almost the same due to difference in spreading.

We also found that the cell area changes depending on the conditions involved. Cells in monolayers tend to be smaller than those that are isolated from one another. This is caused by the lateral pressure from the cells in monolayers, constraining planar spreading and pushing of cells into the third dimension. To reproduce this effect in our 2D study, we assigned different target areas to cells that were exposed to different conditions. The collected cell shape data were used for the development and validation of our model.

### Mathematical model

#### Mathematical model statements

Our imaging studies clearly show that both CMs and FBs have a characteristic polygonal shape. Several studies explain the origin of such shapes as complex biophysical processes, which include formation–destruction of the attachments at the cell boundary, actin polymerisation and the subsequent migration of the cell body^27^. To describe such processes, we proposed the use of the large Potts model. More specifically, we used the GGH model^10–12^, as it is widely applied to reproduce correct cell shapes^28, 29^ and their dynamics^30–32^ in many morphogenetic processes. Our model proffers new rules for the formation and retraction of adhesion sites. We introduced a Hamiltonian with elastic, adhesive and stretching forces, while taking into account the proper description of the interaction between different types of cells and cells with a nanofibrous scaffold. The details of our approach are given below and in the methods section.

#### Basic GGH model

**Figure 3:**
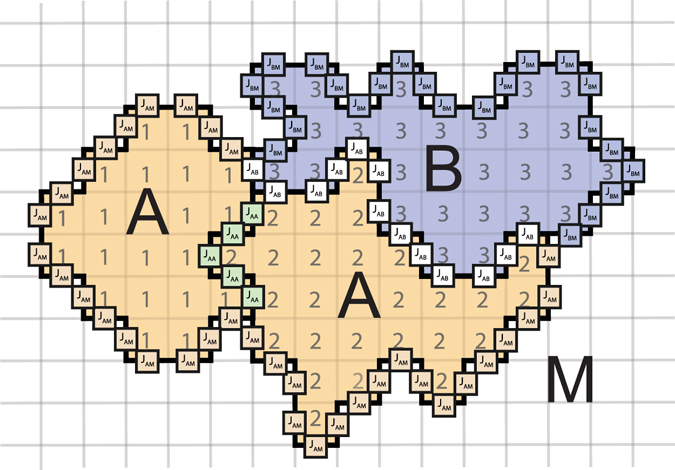
Cells representation in GGH model. Cells of type A are shown in yellow and of type B–in blue. Media around the cells is shown in white and denoted in the model with zero index. Cells interact via adhesion with the surface energy J. This energy depends on the cell types involved (shown in different colours and with different indices).

In GGH modelling (Glazier and Graner, 1992^10^; Graner and Glazier, 1993^11^, Hogeweg, 1997^12^), cells are represented as a cluster of subcells (see Fig. 3) in a regular lattice. Lattice representation allows researchers to efficiently reproduce shapes of the real cells and structures of real tissues. In a basic model, cells maintain their volume and interact with each other via type-specific adhesion which is expressed in terms of the total energy of the system. The strongest part of GGH models is that this energy can be extended with the extra terms to include new forces or fields. The main idea is that cell is allowed to change its shape to minimise the total energy. The process of energy minimization is performed in a way, that it describes not only the static morphology of the cells but also their motility^33^.

Each cell is allowed to change its shape to minimize the prescribed energy. At each time step one of the subcells in a lattice is selected. This subcell attempts to copy itself to a neighbouring position. If this change leads to the decrease of the total energy, it will be accepted. Otherwise, the copy attemts with smaller energy increase are accepted with a higher probability.

This energy-based approach reproduces many static and dynamic cell properties including cells proliferation and migration. Moreover, new phenomena, such as, for example, chemotaxis, could be easily included in a model as a new term in the total energy. In our study, we extended the total energy with the term, that describes cell spreading.

The implementation of GGH is normally organised as follows. Index 0 indicates the *medium*, and positive indices designate individual cells. In our model, we consider two cell types. Thus, τ has three possible values: 0 (medium), 1 (CM) or 2 (FB).

Cell formation is defined by the Hamiltonian of GGH, which has the following form:

1 $$H={H}_{{\rm{adhesive}}}+{H}_{{\rm{elastic}}}=\sum _{\mathop{{\rm{neighbours}}}\limits^{(\overrightarrow{i},\overrightarrow{j})}}{J}_{{\tau }_{{\sigma }_{i}},{\tau }_{{\sigma }_{j}}}(1-\delta ({\sigma }_{i},{\sigma }_{j}))+\sum _{\overrightarrow{i}}{\lambda }_{{\tau }_{{\sigma }_{i}}}{({v}_{{\sigma }_{i}}-{V}_{{\tau }_{{\sigma }_{i}}}^{t})}^{2}$$

where *i* is summed over all lattice points or subcells, ${\sigma }_{i}$ is the index assigned to the *i* ^*th*^ subcell and ${\tau }_{\sigma }$ is a type of cell with index *σ*. *J* is the adhesion energy between cells with indexes ${\sigma }_{i}$ and ${\sigma }_{j}$ of types ${\tau }_{{\sigma }_{i}}$ and ${\tau }_{{\sigma }_{j}}$, and $\delta$ is a Kronecker delta function. In the second term *λ* is the elasticity coefficient and ${V}_{{\tau }_{{\sigma }_{i}}}^{t}$ is the target volume that the cell ${\sigma }_{i}$ maintains. The balance between these two energies determines the curvature of the concave parts of the cell^29^. To simulate the convex parts (or the protrusions), this expression was further extended.

We describe cellular motility by using the iterative Markov chain Monte Carlo (MCMC) algorithm, which attempts to copy an index to a randomly selected lattice point ${\overrightarrow{i}}_{t}$ from a random neighbouring cell ${\overrightarrow{i}}_{s}$. We calculate the change in Hamiltonian for this copy ${\rm{\Delta }}H$, and the new state is accepted with a probability:

$$p=\{\begin{array}{cc}1 & {\rm{i}}{\rm{f}}\,{\rm{\Delta }}{\mathscr{H}}\le 0\\ {{\rm{e}}}^{-\frac{{\rm{\Delta }}H}{T}} & {\rm{i}}{\rm{f}}\,{\rm{\Delta }}{\mathscr{H}} > 0\end{array}$$

where *T* corresponds to motility of the cells. In each Monte-Carlo step (MCS) we perform *N* copy attempts, where *N* is the total number of subcells of the lattice.

The resulting dynamic cell movements mimic the motility and spreading of cells. Questions regarding the time course in the model are addressed in Glazier *et al*. review^33^.

#### Equations and parameters

In our formulation of the GGH model, cell formation is defined by the following Hamiltonian:

2 $$H={H}_{{\rm{adhesive}}}+{H}_{{\rm{elastic}}}+{H}_{{\rm{protr}}}+{H}_{{\rm{nuclei}}}$$

The first two terms in (2) comprise the minimal GGH model, in which, the cells only maintain the target volume and interact via adhesion. *H*_protr_ in (2) is an important new term that we introduced in our model to describe the protrusion at the attachment sites. Protrusion is a very complex biomechanical process that involves attachment, actin polymerisation and biochemical regulation of tension and assembly/disassembly of the actin cytoskeleton. These adhesion sites protrude further and further from the cell body, expanding as much as possible up to a certain length until they suddenly break apart and retract. This sequence of events can be clearly seen in video 7 in Doyle *et al*.^34^. To describe this protrusion process, we assume that the adhesion site repels from the centre of the cell and that the cells can detach from the substrate with some penalty in energy (*P*_detach_).

**Figure 4:**
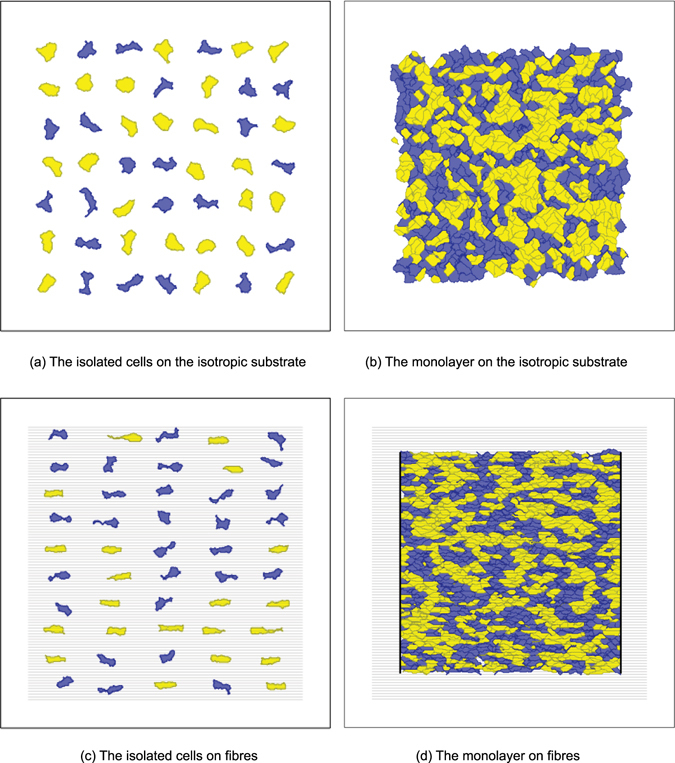
Examples of simulations for four seeding conditions. Cardiomyocytes are shown in yellow tints, whereas fibroblasts are shown in blue tints. Here the proportions of the cells of each type were 50%. Black lines in (**d**) show the borders that constrain the anisotropic monolayer. In (**c**) and (**d**), grey horizontal lines show nanofibres.

We suppose that the cell has a limited number of adhesion sites with protrusive activity (*N*_protr_); all of these repel from the centre of mass (cm). For spreading on the isotropic substrate, we used the simplest possible potential field (*H*_protr_) to describe the expansion of the cell at the attachment sites.

3 $${H}_{{\rm{p}}{\rm{r}}{\rm{o}}{\rm{t}}{\rm{r}}}=\sum _{\mathop{{\rm{f}}{\rm{o}}{\rm{c}}{\rm{a}}{\rm{l}}\,{\rm{c}}{\rm{o}}{\rm{n}}{\rm{t}}{\rm{a}}{\rm{c}}{\rm{t}}{\rm{s}}}\limits^{}}\frac{{G}_{{\tau }_{{\sigma }_{i}}}}{\rho (\overrightarrow{i},{\mathop{{\rm{c}}{\rm{m}}}\limits^{\longrightarrow }}_{{\sigma }_{i}})}+\sum _{{\scriptstyle \begin{array}{c}\mathop{{\rm{f}}{\rm{o}}{\rm{c}}{\rm{a}}{\rm{l}}\,{\rm{c}}{\rm{o}}{\rm{n}}{\rm{t}}{\rm{a}}{\rm{c}}{\rm{t}}{\rm{s}}}\limits^{}\\ \mathop{{\rm{d}}{\rm{e}}{\rm{t}}{\rm{a}}{\rm{c}}{\rm{h}}\,{\rm{f}}{\rm{r}}{\rm{o}}{\rm{m}}\,{\rm{s}}{\rm{u}}{\rm{b}}{\rm{s}}{\rm{t}}{\rm{r}}{\rm{a}}{\rm{t}}{\rm{e}}}\limits^{}\end{array}}}{P}_{{\rm{d}}{\rm{e}}{\rm{t}}{\rm{a}}{\rm{c}}{\rm{h}}},$$

where ${G}_{{\tau }_{{\sigma }_{i}}}$ is the type-dependent constant regulating the amplitude of the protrusion force, and $\rho (\vec{i},{\mathop{{\rm{cm}}}\limits^{\longrightarrow}}_{{\sigma }_{i}})$ is the distance between the currently tested subcell *i* and the centre of mass of the cell. We have chosen the potential as $\mathrm{1/}\rho$, which results in an expansion force that is strong close to the cell, but that decreases with increase in distance from the cell. At some distance, it is balanced by the elasticity term. We found that this representation produces a polygonal form with concave-free arcs in between protrusions (see the discussion related to Fig. 4 later in the text).

Further modifications were needed for the cell–fibre interaction. The fibres in our model occupy some subcells of the mesh and have an assigned orientation. The cells produce internal forces by constructing actin stress fibres from a focal contact towards the cell body. The stronger the response from the scaffold, the more the production of actin filaments, and the higher the total force applied by the cell to the substrate^35, 36^. Assuming that the polymer nanofibres produce a mechanical reaction force only in the direction along them, the energy term for cell–substrate interaction (3) becomes as follows:

4 $$\begin{array}{ccc}{H}_{{\rm{p}}{\rm{r}}{\rm{o}}{\rm{t}}{\rm{r}}} & = & \sum _{\begin{array}{c}\overrightarrow{i}\\ {\rm{f}}{\rm{o}}{\rm{c}}{\rm{a}}{\rm{l}}\,{\rm{c}}{\rm{o}}{\rm{n}}{\rm{t}}{\rm{a}}{\rm{c}}{\rm{t}}{\rm{s}}\\ {\rm{w}}{\rm{i}}{\rm{t}}{\rm{h}}\,{\rm{i}}{\rm{s}}{\rm{o}}{\rm{t}}{\rm{r}}{\rm{o}}{\rm{p}}{\rm{i}}{\rm{c}}\,{\rm{s}}{\rm{u}}{\rm{b}}{\rm{s}}{\rm{t}}{\rm{r}}{\rm{a}}{\rm{t}}{\rm{e}}\end{array}}\frac{{G}_{{\tau }_{{\sigma }_{i}}}}{\rho (\overrightarrow{i},{\mathop{{\rm{c}}{\rm{m}}}\limits^{\longrightarrow }}_{{\sigma }_{i}})}\\  &  & +\sum _{\begin{array}{c}\overrightarrow{i}\\ {\rm{f}}{\rm{o}}{\rm{c}}{\rm{a}}{\rm{l}}\,{\rm{c}}{\rm{o}}{\rm{n}}{\rm{t}}{\rm{a}}{\rm{c}}{\rm{t}}{\rm{s}}\\ {\rm{w}}{\rm{i}}{\rm{t}}{\rm{h}}\,{\rm{n}}{\rm{a}}{\rm{n}}{\rm{o}}{\rm{f}}{\rm{i}}{\rm{b}}{\rm{e}}{\rm{r}}{\rm{s}},\\ {\rm{w}}{\rm{h}}{\rm{e}}{\rm{n}}\,{\rm{o}}{\rm{n}}\,{\rm{n}}{\rm{a}}{\rm{n}}{\rm{o}}{\rm{f}}{\rm{i}}{\rm{b}}{\rm{e}}{\rm{r}}\end{array}}\frac{{G}_{{\tau }_{{\sigma }_{i}}}}{\rho (\overrightarrow{i},{\mathop{{\rm{c}}{\rm{m}}}\limits^{\longrightarrow }}_{{\sigma }_{i}})}{\cos }^{-1}({\phi }_{{f}_{i}}-(\overrightarrow{i},{\hat{\mathop{{\rm{c}}{\rm{m}}}\limits^{\longrightarrow }}}_{{\sigma }_{i}}))\\  &  & +\sum _{\begin{array}{c}\overrightarrow{i}\\ {\rm{f}}{\rm{o}}{\rm{c}}{\rm{a}}{\rm{l}}\,{\rm{c}}{\rm{o}}{\rm{n}}{\rm{t}}{\rm{a}}{\rm{c}}{\rm{t}}{\rm{s}}\\ {\rm{d}}{\rm{e}}{\rm{t}}{\rm{a}}{\rm{c}}{\rm{h}}\,{\rm{f}}{\rm{r}}{\rm{o}}{\rm{m}}\,{\rm{s}}{\rm{u}}{\rm{b}}{\rm{s}}{\rm{t}}{\rm{r}}{\rm{a}}{\rm{t}}{\rm{e}}\end{array}}{P}_{{\rm{d}}{\rm{e}}{\rm{t}}{\rm{a}}{\rm{c}}{\rm{h}}}+\sum _{\begin{array}{c}\overrightarrow{i}\\ {\rm{f}}{\rm{o}}{\rm{c}}{\rm{a}}{\rm{l}}\,{\rm{c}}{\rm{o}}{\rm{n}}{\rm{t}}{\rm{a}}{\rm{c}}{\rm{t}}{\rm{s}}\\ {\rm{d}}{\rm{e}}{\rm{t}}{\rm{a}}{\rm{c}}{\rm{h}}\,{\rm{f}}{\rm{r}}{\rm{o}}{\rm{m}}\,{\rm{n}}{\rm{a}}{\rm{n}}{\rm{o}}{\rm{f}}{\rm{i}}{\rm{b}}{\rm{e}}{\rm{r}}\end{array}}{P}_{{\rm{u}}{\rm{n}}{\rm{l}}{\rm{e}}{\rm{a}}{\rm{s}}{\rm{h}}},\end{array}$$

where for movements along the fibres, the projection of the force to the nanofibre is considered. Given that the cell builds up new actin filaments to counteract the tension along the nanofibre, the projection of the distance *r*cos(*α*) instead of the distance *r* itself was used (see Section III C for more details). $(\vec{i},{\widehat{\mathop{{\rm{cm}}}\limits^{\longrightarrow}}}_{{\sigma }_{i}})$ denotes the direction of the vector from the centre of mass ${\mathop{{\rm{cm}}}\limits^{\longrightarrow}}_{{\sigma }_{i}}$ to the currently examined subcell $\mathop{i}\limits^{-}$. The difference between this direction and fibre direction (*α* in the description above) is used for projection calculation.

To describe the interaction of the attachment sites with the nanofibre, we assume that movements from the isotropic substrate to the fibre require no energy change. In our experiments, we covered the isotropic and anisotropic monolayers with the same fibronectin solution, so that integrins at the cell surface bound to the fibronectin the same way. Therefore, we conclude, that there is no difference in adhesive properties between the nanofibres and the isotropic substrate. However, for movements from the fibre back to the isotropic substrate, we apply the penalty *P*_unleash_. It is required because our experimental observations show that the cell builds up actin filaments during motion along the nanofibres. These filaments have to be aligned with the nanofibres. However, the motion from the fibre back to the isotropic surface is the motion aside from that in the actin filament direction that should be performed by a stress fibre not aligned with the nanofibre. Thus, such motion is energetically unfavourable in terms of our model. As a result, cell elongation occurs because of actin strand reassembly, which is controlled by the direction-dependent mechanical reaction of the substrate. However, the same forces also create direction-dependent motility, which impose constraints on the monolayer from the sides because of the high motility along the fibres.

One more penalty term exists in our model:

$${H}_{{\rm{nuclei}}}=\sum _{i}\,{P}_{{\rm{N}}}$$

for the invasion of the media (index 0) or the other cell deep into the current cell. We assume that the cell nucleus and the surrounding area have a higher stiffness than the remaining cell body. This penalty term *P*_*N*_ has a non-zero value for the extraneous subcells close to the nucleus.

Finally, three more rules for copy attempts in our model are not present in the energy equation. The copy is forbidden in three cases: if, as a result, a cell disappears; if the connectivity of the cell breaks; or if the protrusion spreads further than *L*_MAX_ from the cm.

The resulting model is described in detail in section III C.

#### Model validation

**Figure 5:**
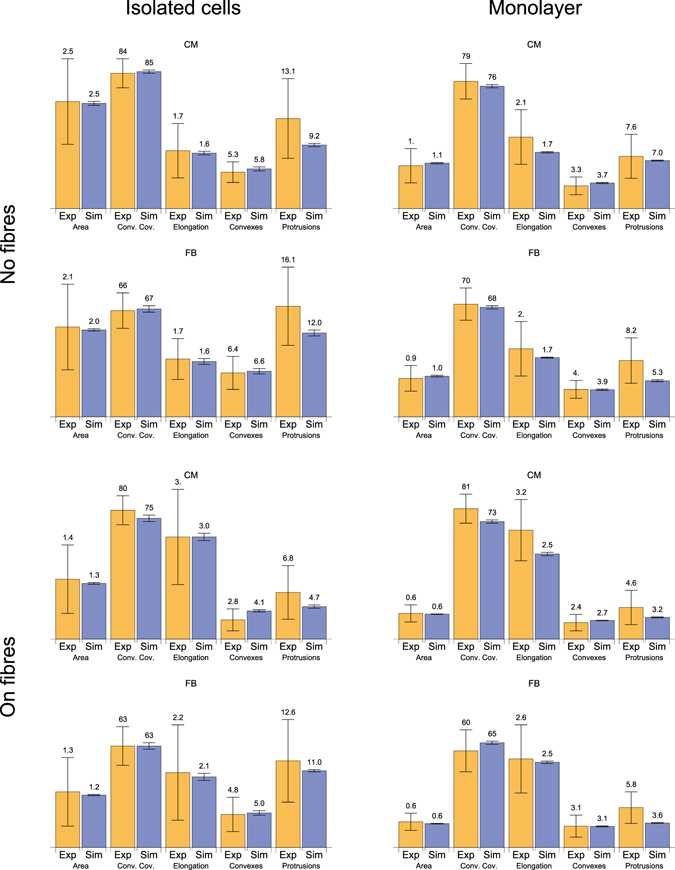
Comparison of the parameters in the experiment and the simulation. Yellow bars show experimental values, whereas the blue ones show computed values. The amount of cells studied in experiment and considered in this statistics are listed in Table 1, in simulations–in Table 2. Error bars indicate standard deviations.

For each of the four conditions described above, we identified the best set of parameters that fit the experimental shape features. All the parameters for the model are listed in Table 2. The shape characteristics are compared with those in the experimental results in Fig. 5. One can see that most characteristics of the isolated cells almost precisely match the experimental data, except for a number of protrusion sites on the cell periphery. This number was slightly fewer in the simulations but was within the variability of the experimental measurements. For the CMs in monolayers, both with or without fibres, there was also some divergence in elongation, which stays within the deviations in the experiments.

**Table 2:** Parameters of the model.

Parameter	Unit	No fibres				On fibres			
		Isolated cells		Monolayer		Isolated cells		Monolayer	
		CM	FB	CM	FB	CM	FB	CM	FB
Temperature T		1.0		1.0		1.0		1.0	
*G*_N_	*mm*	47.48	26.81	51.03	5.09	238.22	9.62	461.36	233.76
*V*_*t*_	10^3^ *μm* ^2^	2.11	1.39	0.88	0.79	1.34	0.93	0.6	0.35
λ	*mm* ^−4^	151.37	70.71	62.32	17.91	69.88	68.05	26.42	14.24
*P*_detach_	*mm* ^*−1*^	9.89	12.3	30.93	11.22	16.16	15.2	155.62	53.21
*J*_Cell-MD_		427.82	306.96	1013.93	445.77	474.19	305.8	937.13	560.27
*J*_Cell-Cell_		—	—	798.73	473.28	—	—	631.42	267.25
*J*_CM-FB_		—		949.22		—		1152.05	
*P*_unleash_		—	—	—	—	28.15	1.44	117.94	66.93
*L*_MAX_	*μ*m	66.64	76.7	81.41	73.62	42.31	48.72	62.37	65.05
*N*_protr_(fixed)		21	24	12	13	10	22	8	9
Sample dim.	mm × mm	1.0 × 1.0		0.8 × 0.8		1.0 × 1.0		0.8 × 0.8	
Simulation time	MCS	900		2000		2000		3000	
Number of cells	1	7 × 7		26 × 26		5 × 10		17 × 68

Although the average values in the model and in the experiment are in good agreement, there is a substantial difference in the variability of most of the shape parameters: the variability in the experiments exceeds that in the simulations. The main reason is the homogeneity of the cell population considered. However, in the experimental samples, even adjacent cells may have different basic parameters, such as exclusive volume and generation of protrusion force. This discrepancy can be overcome by shifting beyond a single parameter set paradigm and applying the approach of experimentally calibrated populations of models; this approach is now under development in cardiac electrophysiology^37, 38^. However, in the first step, we decided not to include cell variability. Overall, we conclude that our model has a good correspondence with the properties of the average cell measured in the experiment.

The resulting computed shapes of the cells for each of the cases are shown in Fig. 4. Visually, the shapes also correspond well with the experimental data, apart from the agreement found in the statistical evaluation.

### Wave propagation in isotropic and anisotropic samples with fibrosis

Using the developed morphological models, we further studied wave propagation in fibrotic tissue. Here, we aimed to reproduce such properties of the experimental tissue as velocity, anisotropy and wavefront complexity. We tested wave propagation in isotropic and anisotropic virtual tissue and compared the results with those of the optical mapping of the experimental cardiac monolayers.

Initially, we measured the concentration of FBs in the experimental sample by using immunohistochemical data. Next, this number was used for virtual tissue generation with our GGH model. Finally, for the wave propagation simulations, we applied a monodomain ionic model of neonatal rat ventricular CM, developed by R. Majumder in 2016^39^. This model is a modification of the Kohronen model with simplified calcium dynamics. We used the mesh generated by our GGH model for our electrophysiological simulations. Different coupling coefficients were assigned to propagation along the cell, end-to-end and side-to-side signal transmission between the cells. The difference between end-to-end and side-to-side connections was related to the non-uniform GJs and ionic channels distribution along the cell membrane. These coefficients were adjusted to fit the experimental velocities.

**Figure 6:**
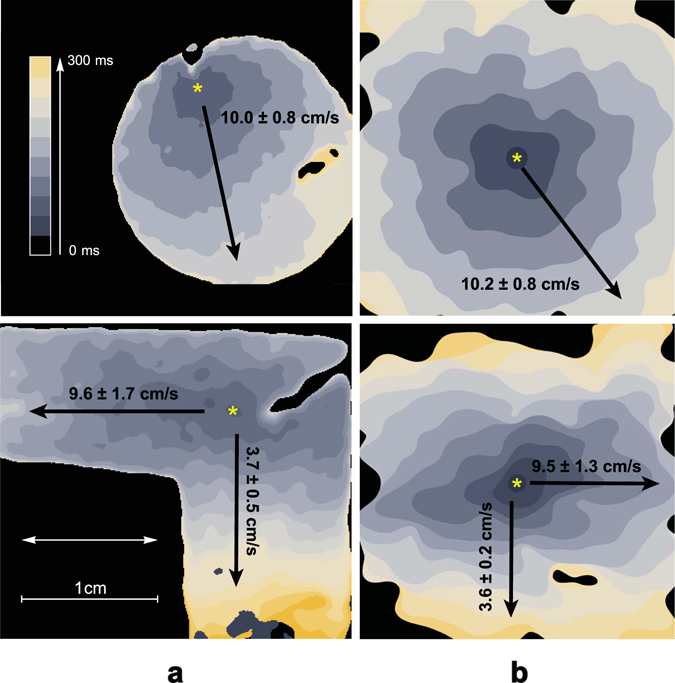
Experimental results acquired with the optical mapping of isotropic and anisotropic samples compared to simulated ones. (**a**) Activation maps for the isotropic sample (upper image) and the anisotropic sample on nanofibers (lower image). The yellow star shows the place where stimulation with an electrode was applied. The white arrow shows the preferred direction of the fibres in the sample. Activation time is colour coded (0–300 ms). (**b**) Corresponding simulations of the wave propagation in samples with 30% and 35% FBs in isotropic and anisotropic samples, respectively. The same filters with the same kernel size were applied to the simulated data, as for the experimental data in column (**a**). Corresponding video files and extended version of this figure could be found in Supplementary Materials.

Figure 6 shows the activation maps for the experimental samples (left) and simulations (right). The yellow star indicates the place where the stimulations were applied, and the white arrow shows the nanofibre orientation. The arrival time is colour coded. One can see that both in the experiment and the simulation, the wavefronts have a similar shape complexity.

In isotropic tissue (without nanofibers) on the large scale, the velocity ratio was always 1 regardless of cell elongation and non-uniform channels distribution along the cell membrane. In the presence of nanofibres, we observed anisotropy. To measure the anisotropy more precisely, we prepared L-shaped experimental samples. These samples were covered with nanofibres aligned along one of the arms of the L. We stimulated the corner of the L-sample and measured the longitudinal and transversal velocities in the corresponding arms. This shape of the sample allowed us to avoid the effects of curvature on wavefront velocity. The measured velocity ratio for the anisotropic samples varied between 1.4 and 2.6 (in 8 samples, where 5 samples were made of the cells from one isolation). In Fig. 6 the sample with the highest anisotropy ratio is shown.

In the simulations with 35% of FBs, the velocity ratio stayed within a range between 1.5 and 2.7 depending on the parameters of the electrophysiological model. This range was determined by tissue morphology and was similar to the observed range of anisotropy ratios in experimental samples. Both in simulation and experiment, zig-zag propagation occurred along the transversal direction. This can be seen in the activation map: there are regions with the vertical wavefront direction, perpendicular to the overall propagation direction. The corresponding video of wave propagation could be found in Supplementary Material. This zigzag propagation resulted in high anisotropy ratio.

Taken together, using our approach, we reproduced the proper anisotropy for the samples on nanofibres, as well as a similar wave complexity for the wavefronts both in isotropic and anisotropic cases. Therefore, we have shown that our morphology-based model provides a detailed and accurate description of wave propagation and captures wavefront complexity.

## Discussion

The morphology of cardiac tissue significantly influences its function. It is well known, for example, that with ageing, CMs become larger, and the number of FBs increases, filling the interstitial spaces between CM bundles. The conduction velocity then decreases, and the functional anisotropy increases^40^, resulting in a proarrhythmic substrate. Thus, understanding the connection between these functional and morphological changes is important.

In this study, we developed a powerful tool to study the relation between morphology and electrical wave propagation in cardiac monolayers. Using our approach, we demonstrated the possibility to accurately reproduce cell shapes under various conditions, functional anisotropy and complexity of the wavefronts. We collected a database of cell shapes under different conditions: low density seeding, high density seeding, with and without nanofibres. We selected and measured the characteristic parameters for these shapes and found that FBs can be recognised in cardiac tissue by their shape alone. This is due to the fact that FBs tend to have regions of deeper concavity on the cell boundary than CMs. We proposed a model to describe these features and adjusted to fit experimental data. Our model is based on the GGH model^10–12^, which is widely used in tissue growth studies. After adjustments, our model could accurately reproduce cell shape parameters in four studied cases. Interestingly, the virtual cells on the isotropic substrate exhibited a slight elongation to the ratio of 1.7. Furthermore, our model does not include mechanisms for cell polarisation. Any observed polarisation was solely caused by the process of cell spreading at a discrete number of attachment sites.

We used the virtual cell culture generated with our model for electrophysiological simulations. A resulting wave propagation pattern accurately reproduced the experimentally observed behaviour for both isotropic and anisotropic cases. In the isotropic case, despite the high elongation of virtual cells, the resulting excitation propagation was also isotropic as in the experiment due to the lack of preferential direction^41–43^. For the anisotropic monolayer, a correct range of anisotropy ratios and wavefront complexity were achieved. An overview on our study is presented in Figure S1 in Supplementary Material.

Our GGH-type model for cardiac tissue describes discrete biological cells. Several groups have already proposed discrete models of different designs, which were mainly used to study the discontinuous nature of cardiac propagation. The first discrete model was proposed by Spach *et al*.^44^ and contained 33 adult coupled cells, replicated directly from the experimental images. This model explained the fact that the maximum rate of rise of the transmembrane potential (*V*_*max*_) depends on the direction of propagation, which was previously found in the experiment^45^. Henriquez *et al*.^46^ demonstrated the role of cellular shape irregularities in a conduction block and in re-entry formation; they also introduced FBs to the model^47^ and studied how various coupling coefficients would affect wave propagation. In these studies, randomised cell forms were generated by a custom iterative growth algorithm^48^ that was adjusted to fit length and width distributions. Since cell shapes in their studies were irregular, they were triangulated and FEM algorithm for electrical simulations was applied (in 2D^49^ and 3D^50^). Other groups that encountered anisotropic and discontinuous effects also developed discretisation algorithms similar to Henriquez^51, 52^. From this perspective, we propose a discrete model of a new kind; it combines both realistic cell shapes of the Spach model and the scalability of the models of Jacquemet-Henriquez^47^, Lin-Keener^52^ and Prudat-Kucera^51^.

FBs are ubiquitous in cardiac tissue and are indispensable in engineered cardiac cell cultures^53^. Thus, we also included FBs into our morphological model. With additional improvements in our model describing the spatial distribution of FBs in a culture, it would be possible to study the arrhythmogenicity of different types of fibrosis. Depending on the parameters, samples with patchy, diffuse or interstitial fibrosis could be generated. Such modelling may provide some ideas on fibrosis development and possible ways of treatment.

Note that currently, our monolayer formation model is only at the first stage of development. However, various general scientific questions can already be addressed with it. For example, it can be applied to study the development of iPS cell cultures *in vitro* or cell clusters *in vivo*, which is one of the hottest topics in cardiac stem cell research^54^. It can be used to study percolation phenomena in cardiac tissue, which is considered an important determinant of atrial fibrillation^55^. More generally, such a model can be applied to describe the dynamic remodelling of cardiac tissue after myocardial infarction or during the development of heart failure. Additionally, the electrophysiological model may be further improved. Using our virtual tissues, one can study various types of cell-to-cell communications. It may include study of the role of the non-uniform distribution of ionic channels in cell membranes and non-junctional cell-to-cell signal transmission. However, the further development of our approach is needed to achieve all these goals.

Firstly, additional research related to cell migration and dynamics is required. The current version of our model, adjusted to the experiment, exhibits low motility of the cells. However, migration is a very important part of tissue development that provides cell sorting and clustering. Cell clustering may then substantially change propagation safety and anisotropy. Switching the migration on in our model is possible by increasing motility by decreasing the penalty for detachment and target spreading areas. As the result of this change, cells would be able to migrate and sort, but not able to spread properly. Probably, these parameters should evolve during monolayer formation, allowing migration at the first stage and spreading at the second stage. However, additional time-lapse data regarding monolayer formation are required to develop a valid dynamic model for monolayer formation.

Next, the cell shapes in experimental cell cultures vary greatly according to our experimental data. Our model, however, reproduces only the average cell, but not the ensemble of cells with a wide parameter distribution. The dynamics of the real cells observed in the experiment may be unique for each cell. The parameters of our model should be modified in a way that each cell has its own target volume, protrusion dynamics, and other parameters. This approach creates a family of models and was already successfully implemented in cardiac electrophysiology. It describes experimentally observed AP variability^38^ and modulation^56^ and can be also extended to describe morphological variations between the cells in the tissue.

In this paper we apply discrete modelling approach to reproduce the complexity of the cardiac microstructure. Alternatively, the heterogeneous tissues could be described with the modern continuous models^56–59^. In these models, the continuous diffusion term is substituted by a fractional Laplacian^57, 56^ or porous-medium diffusion term^58^ to account for high heterogeneity of the tissue. Discrete and continuous approaches could efficiently complement each other. For example, one could perform homogenisation for our discrete model and verify the resulting excitation patterns. The continuous model could be then used for large-scale 3D simulations of the real heart, still taking into account the discontinuous nature of the wave propagation in heterogeneous media.

Finally, the model can be extended to a third dimension to describe *in vivo* tissues. Doing so can allow us to reproduce the observed changes in the area of the isolated cells compared with the cells in cultures in terms of single parameter sets, and to generate proper cell elongation more easily. This is because in our model, cell extension was prevented by the pressure arising from the interaction of a given cell with its neighbouring cells. This effect will be absent in 3D because the cell would be able to move into the third dimension by increasing its height. Including real ECM^60^ in a model and studying its guiding role in cardiac tissue formation would also be possible.

## Methods

### Experimental samples preparation

#### Neonatal cardiac cell isolation

All studies conformed to the Guide for the Care and Use of Laboratory Animals, published by the United States National Institutes of Health (Publication No. 85-23, revised 1996) and approved by the Moscow Institute of Physics and Technology Life Science Center Provisional Animal Care and Research Procedures Committee, Protocol #A2-2012-09-02. In this study, we used enzymes adapted to the existing two-day protocol selection from Worthngton-Biochem. (http://www.worthingtonbiochem.com/NCIS/default.html). Cardiac cells were isolated from the ventricles of rat pups (Rattus norvegicus, Sprague Dawley breed) with different ages (1–4 days). Then, the isolated cells were seeded on the specimens covered with fibronectin (0,16 mg/ml, Gibco, USA, 33016015) at different concentrations before they were cultivated in DMEM culture medium (Gibco, USA, 11960) with 5% of FBS (foetal bovine serum, Gibco, USA, 10100147). For the study of the shapes of the isolated cells, the cells were seeded at 5 · 10^3^ cells/cm^2^. After 3 days of cultivation, the samples were fixated. The monolayers of primary culture cells were seeded at 30 · 10^3^ cells/cm^2^, and after 3–5 days, the monolayers were confluent, performed coordinated contraction activity and, therefore, were used in morphometrical studies and optical mapping.

#### Nanofibre preparation

The polycaprolactone (PCL) solution was prepared by dissolving PCL powder (Sigma-Aldrich, USA, 440744) in hexafluoroisopropanol at concentrations of 10%–15%. The prepared solution was electrospun with Nanon-01 electrospinning setup (MECC CO., LTD), with the applied voltage between the syringe tip and the grounded collector in the range of 5 kV to 10 kV. It was loaded into the 3 ml syringe and ejected through the 20 gauge blunt tip needle at a flow rate of 0.1–1 ml/h with the use of a programmable syringe pump (Fusion 100, Chemyx Inc., Japan). Nanofibres were electrospun directly onto the surface of a 15 mm-diameter cover glass deposited on the grounded collector. The angular velocity was 1,000 rpm. After the electrospinning process was completed, the non-adhesive PCL nanofibrous substrates were coated with a solution of human plasma fibronectin (0.16 mg/ml in PBS, Gibco, USA, 33016015) to produce a cell adhesive matrix.

Note that only one fibre density was used in all experiments for both monolayer and isolated cell setups.

#### Immunohistochemical staining

The cells were fixated with 5% PFA (paraformaldehyde powder, 95%, Sigma-Aldrich, USA, 158127-100 G), and nuclear staining was performed with DAPI (VECTASHIELD Mounting Medium with DAPI, Vector, USA, Cat. No. H-1200). In our work, we used anti-*α*-actinin (Sigma-Aldrich, USA, A7811)and alexa fluor 594 like Secondary Antibody (A-11020, Life Technologies) for CM-specific labelling, Alexa Fluor 488 phalloidin (Molecular Probes, USA, A12379) for F-actin non-specific staining and DAPI for labelling cell DNA. Pictures were taken with an inverted fluorescence microscope (Axio Imager with ApoTome optical sectioning module, Zeiss). Immunofluorescent staining of the CMs was performed with the use of secondary and primary antibodies according to a previously described protocol (http://www.abcam.com/protocols/immunocytochemistry-immunofluorescence-protocol).

#### Optical mapping

To monitor activity and record the excitation patterns, the 3- to 5-day-old monolayers were loaded with the Ca^2+^ -sensitive indicator Fluo-4-AM (Molecular Probes, USA, F14201). After staining, the medium was exchanged with Tyrode’s solution (Sigma-Aldrich Co., USA, T2145-10L) and kept at room temperature during the observations. The excitation waves were monitored with a high-speed imaging setup (Olympus MVX-10 Macro-View fluorescent microscope equipped with high-speed Andor EM-CCD Camera 897-U at 68 fps).

All videos were processed with ImageJ software. The experimental optical mapping signal was processed for noise reduction and better wavefronts representation. The Gaussian smoothing filter was applied with 250*μm* and 300*μm* kernel size for isotropic and anisotropic samples respectively. The same processing with the same kernel size was applied to the simulated waves (see Fig. 6).

#### Velocity ratio measurements for anisotropic tissue

The L-shaped samples were prepared for velocity ratio measurements (see Fig. 6). The nanofibres were spinned parallel to one of the arms. The electrode was placed at the joint part, at the intersection of the middle lines of the arms. As a result, both arms were stimulated equally. The longitudinal and transversal velocities were measured for the plane wavefronts propagating along each of the arms.

### Cell shape acquisition

Neonatal rat cells were stained with DAPI (DNA, blue), phalloidin (F-actin, green) and monoclonal anti-*α*-actinin antibody (*α*-actinin, red). Phalloidin staining was also compared with the optical images to ensure that F-actin fibres highlight the edge of the cell (not shown). From the *α*-actinin image, CMs were discriminated from FBs (see Fig. 2). For the F-actinin images, the contrast was enhanced so that the whole cells were coloured. Then, for the isolated cells, we selected the filled area and manually included all the small inner parts that were not enriched with F-actin and, therefore, not selected automatically. For CMs in monolayers surrounded by FBs, the same technique with *α*-actinin staining was used. As a result, each CM was highlighted separately. In the other context in monolayers, we had to outline the cell shape manually. Following this procedure, the database of cell shapes was collected for each of the four conditions that we studied (see Fig. 1). After the cell was selected, the contour was transferred to the vector form, and the obtained curve was then used in the shape analysis.

All processing was done with custom Wolfram Mathematica (WM) (http://www.wolfram.com/mathematica/?source=nav) code using a ComponentMeasurements[] tool. All possible parameters related to cell shape were checked. Those that are either necessary for the model or characteristic of one of the cell types were selected. Firstly, the total area and elongation were measured. Elongation here is the ratio between the largest and the smallest diameter of the convex hull (“Caliper Elongation”). In WM, the elongation is defined as 1−*w*/*l*, where *w* is the width and *l* is the length. We changed this definition to *l/w*, which is more useful in our study. Next, the cell was surrounded with a convex hull with the use of the “Convex Vertices” parameter of ComponentMeasurements[] in WM. The proportion of the convex hull occupied by the cell appeared to be an important parameter and was substantially different for CMs and FBs. Therefore, the ratio of the cell area and the area of the convex hull was also included in the list of parameters.

Cardiac cells have a characteristic polygonal shape because they have a discrete number of adhesion sites that are essential for the spreading process. The number of these adhesion sites can be found through observation of the F-actin image. Actin filaments are concentrated in these mature adhesion sites pulling the cell body towards this point. From the images, we could count the number of adhesion sites manually. However, we also developed an automatic algorithm to determine the quantity of adhesion points. It is based on the fact that protrusive activity at the adhesion sites results in convexity formation. To count the convex regions, we rasterised cell contours with a resolution of 1.0*μm* per pixel and applied skeleton transform to it (using SkeletonTransform[] in WM). The raw experimental data had a different but higher resolution (of 0.10–0.42*μm*/px). Then, the central part of the skeleton transform was removed, and the number of tips was counted. This number of protrusions was compared with the number of adhesion sites, which were counted manually (for CMs ${N}_{{\rm{CM}}}^{{\rm{pr}}}=18\pm 4$ (*n*_CM_ = 36), for FBs ${N}_{{\rm{FB}}}^{{\rm{pr}}}=21\pm 5$ (*n*_FB_ = 45)), and the algorithm gave similar results (for CMs ${N}_{{\rm{CM}}}^{{\rm{pr}}}=14\pm 5$ (*n*_CM_ = 36), for FBs ${N}_{{\rm{FB}}}^{{\rm{pr}}}=16\pm 6$ (*n*_FB_ = 45)).

However, the space resolution of our model was chosen to be lower (2.5 *μ*m instead of 1.0 *μ*m). Such a space step of 2–2.5 *μ*m is standard in various GGH models for most eukaryotic cell simulations^13, 14, 61^ and electrophysiological studies in discrete cellular models^51^. For this resolution, some of the protrusions average out and are not pronounced enough to be detected with the skeleton transform algorithm. As a result, the number of convexes in our model turned out to be less than that in the experiment. However, some parts of the discrete border of the cell in the simulation may also be recognised as an additional protrusion. Despite the possible misinterpretation of the results of this algorithm, we suggest that it is appropriate for the comparison of the computed cell shapes with the experimental cell shapes. However, the algorithm in both cases should be applied to the images rescaled to the same resolution.

#### Statistics for convex coverage

Data are expressed as mean ± standard deviation (SD). Analysing the parameters listed in Table 1, it was found that FBs have much deeper concaves than CMs, resulting in a less convex coverage (see Fig. 2). CMs normally occupy around 80% of the convex hull, whereas FBs occupy only 60–70% of it. The distributions of the convex coverage over the cell population were compared and Pearson’s chi-squared test was applied to determine whether the medians of these distributions are equal. The *p*-value for this hypothesis was <10^−3^ (*n*_CMs_ = 36, *n*_FBs_ = 45), which means that it is very unlikely that these distributions have the same median value. The outcome of other seeding conditions is analogous.

### Data availability

The data that support the findings of this study are available from the corresponding authors upon reasonable request.

### Mathematical model

Cell formation is defined by the Hamiltonian of the GGH model, which, in our model, is equal to the following:

5 $$\begin{array}{ccc}H & = & \sum _{\begin{array}{c}(\overrightarrow{i},\overrightarrow{j})\\ {\rm{n}}{\rm{e}}{\rm{i}}{\rm{g}}{\rm{h}}{\rm{b}}{\rm{o}}{\rm{u}}{\rm{r}}{\rm{s}}\end{array}}{J}_{{\tau }_{{\sigma }_{i}},{\tau }_{{\sigma }_{j}}}(1-\delta ({\sigma }_{i},{\sigma }_{j}))+\sum _{\overrightarrow{i}}{\lambda }_{{\tau }_{{\sigma }_{i}}}{({v}_{{\sigma }_{i}}-{V}_{{\tau }_{{\sigma }_{i}}}^{t})}^{2}\\  &  & \,+\sum _{\begin{array}{c}\overrightarrow{i}\\ {\rm{f}}{\rm{o}}{\rm{c}}{\rm{a}}{\rm{l}}\,{\rm{c}}{\rm{o}}{\rm{n}}{\rm{t}}{\rm{a}}{\rm{c}}{\rm{t}}{\rm{s}}\\ \,{\rm{w}}{\rm{i}}{\rm{t}}{\rm{h}}\,{\rm{g}}{\rm{l}}{\rm{a}}{\rm{s}}{\rm{s}}\end{array}}\frac{{G}_{{\tau }_{{\sigma }_{i}}}}{\rho (\overrightarrow{i},{\mathop{{\rm{c}}{\rm{m}}}\limits^{\longrightarrow }}_{{\sigma }_{i}})}\\  &  & \,\,+\sum _{\begin{array}{c}\overrightarrow{i}\\ {\rm{f}}{\rm{o}}{\rm{c}}{\rm{a}}{\rm{l}}\,{\rm{c}}{\rm{o}}{\rm{n}}{\rm{t}}{\rm{a}}{\rm{c}}{\rm{t}}{\rm{s}}\\ \,{\rm{w}}{\rm{i}}{\rm{t}}{\rm{h}}\,{\rm{f}}{\rm{i}}{\rm{b}}{\rm{e}}{\rm{r}}\end{array}}\frac{{G}_{{\tau }_{{\sigma }_{i}}}}{\rho (\overrightarrow{i},{\mathop{{\rm{c}}{\rm{m}}}\limits^{\longrightarrow }}_{{\sigma }_{i}})}{\cos }^{-1}({\varphi }_{{f}_{i}}-(\overrightarrow{i},{\hat{\mathop{{\rm{c}}{\rm{m}}}\limits^{\longrightarrow }}}_{{\sigma }_{i}}))\\  &  & +\sum _{\begin{array}{c}\overrightarrow{i}\\ {\rm{f}}{\rm{o}}{\rm{c}}{\rm{a}}{\rm{l}}\,{\rm{c}}{\rm{o}}{\rm{n}}{\rm{t}}{\rm{a}}{\rm{c}}{\rm{t}}{\rm{s}}\\ {\rm{d}}{\rm{e}}{\rm{t}}{\rm{a}}{\rm{c}}{\rm{h}}\,{\rm{f}}{\rm{r}}{\rm{o}}{\rm{m}}\,{\rm{s}}{\rm{u}}{\rm{b}}{\rm{s}}{\rm{t}}{\rm{r}}{\rm{a}}{\rm{t}}{\rm{e}}\end{array}}{P}_{{\rm{d}}{\rm{e}}{\rm{t}}{\rm{a}}{\rm{c}}{\rm{h}}}+\sum _{\begin{array}{c}\overrightarrow{i}\\ {\rm{f}}{\rm{o}}{\rm{c}}{\rm{a}}{\rm{l}}\,{\rm{c}}{\rm{o}}{\rm{n}}{\rm{t}}{\rm{a}}{\rm{c}}{\rm{t}}{\rm{s}}\\ {\rm{f}}{\rm{r}}{\rm{o}}{\rm{m}}\,{\rm{n}}{\rm{a}}{\rm{n}}{\rm{o}}{\rm{f}}{\rm{i}}{\rm{b}}{\rm{e}}{\rm{r}}\end{array}}{P}_{{\rm{u}}{\rm{n}}{\rm{l}}{\rm{e}}{\rm{a}}{\rm{s}}{\rm{h}}}+\sum _{i}{P}_{{\rm{N}}}\end{array}$$

where *i* is summed over all lattice points or subcells, ${\sigma }_{i}$ is the index in the ${i}^{th}$ subcell and ${\tau }_{\sigma }$ is a type of cell with index $\sigma$. In our model, we consider two cell types, so $\tau$ has three possible values: 0 (medium), 1 (CM) and 2 (FB). In the simplest GGH model, the cells are only maintaining the target volume and interacting via adhesion. The first term of the equation (5) is an adhesive energy term given by the following:

$$J=(\begin{array}{ccc}{J}_{\text{MD}{\textstyle \text{-}}\text{MD}} & {J}_{\text{CM}{\textstyle \text{-}}\text{MD}} & {J}_{\text{FB}{\textstyle \text{-}}\text{MD}}\\ {J}_{\text{CM}{\textstyle \text{-}}\text{MD}} & {J}_{\text{CM}{\textstyle \text{-}}\text{CM}} & {J}_{\text{CM}{\textstyle \text{-}}\text{FB}}\\ {J}_{\text{FB}{\textstyle \text{-}}\text{CM}} & {J}_{\text{CM}{\textstyle \text{-}}\text{FB}} & {J}_{\text{FB}{\textstyle \text{-}}\text{FB}}\end{array}),$$

where ${J}_{X \mbox{-} Y}$ is the energy of interaction of the cell of type X with the cell of type Y. The value of ${J}_{\text{MD} \mbox{-} \text{MD}}$ is irrelevant because the whole medium is one subcell with one index zero, and *J*s are applied only on the sub-domain boundaries. Let us then assume that *J*_MD-MD_ = 0. Five independent energies are left, and these determine the matrix *J*. Let us first discuss their relations.

Showing the connection between *J* and surface tension ${s}$ is easy because it basically corresponds to the surface energy. For the cell medium, *s*_CM_ = *J*_CM-MD_, and for the cell–cell surface, ${{s}}_{C \mbox{-} C}={J}_{C \mbox{-} C}\mathrm{/2}-{J}_{C \mbox{-} \text{MD}}$, where ${\rm{C}}$ may be either CM or FB. If ${J}_{C \mbox{-} C} < 2{J}_{C \mbox{-} \text{MD}}$, then the cells will tend to clusterise, and in the opposite case, they will repulse one another. The relation between ${J}_{\text{CM} \mbox{-} \text{CM}}$ (or ${J}_{\text{FB} \mbox{-} \text{FB}}$) and ${J}_{\text{CM} \mbox{-} \text{FB}}$ governs cell sorting and rearrangements^11^. If the energy for one of the cell types (${J}_{\text{CM} \mbox{-} \text{CM}}$ or ${J}_{\text{FB} \mbox{-} \text{FB}}$) is substantially smaller than ${J}_{\text{CM} \mbox{-} \text{FB}}$, then the cells of this type will condense into clusters. In this study, we use these energies as one of the many fitting parameters to adjust the model for cell shape reproduction. However, the rearrangements and clusterisation in cardiac tissue should be further studied in the future.

Generalised cells in the GGH model also have such parameters as volume ${v}_{{\sigma }_{i}}$ and centre of mass cm. The volume of the cell tends to converge to the target volume ${V}_{{\tau }_{{\sigma }_{i}}}^{t}$, and ${\lambda }_{{\tau }_{{\sigma }_{i}}}$ characterises the cell elasticity around that value. The target volumes and elasticity are different for various cell types so that both of them are dependent on type ${\tau }_{{\sigma }_{i}}$.

The next term in (5) describes the protrusion at the attachment sites. We assume that the cell has a limited number of adhesion sites with protrusive activity (*N*_protr_), and their dynamics in the absence of nanofibres are controlled by the potential ${G}_{{\tau }_{{\sigma }_{i}}}/\rho ({\sigma }_{i},{\rm{cm}}({\sigma }_{i}))$, where $\rho ({\sigma }_{i},{\rm{cm}}({\sigma }_{i})$ is the distance from the cm to a given subcell. We proposed the simplest possible potential field to describe the expansion of the cell at the attachment sites, and we added a corresponding term to the GGH model (see Equation (3) in Section I B).

The focal adhesion can be detached from the substrate with a penalty *P*_detach_. It is applied in an attempt to copy a subcell (with a focal adhesion or not) on the existing focal adhesion. If it is detached, then the number of adhesions of the cell to the substrate is reduced. The total number of focal adhesions for a cell should not exceed *N*_protr_. If it is less than *N*_protr_, then during the next successfull expansion of the cell border, a new focal adhesion will be established.

**Figure 7:**
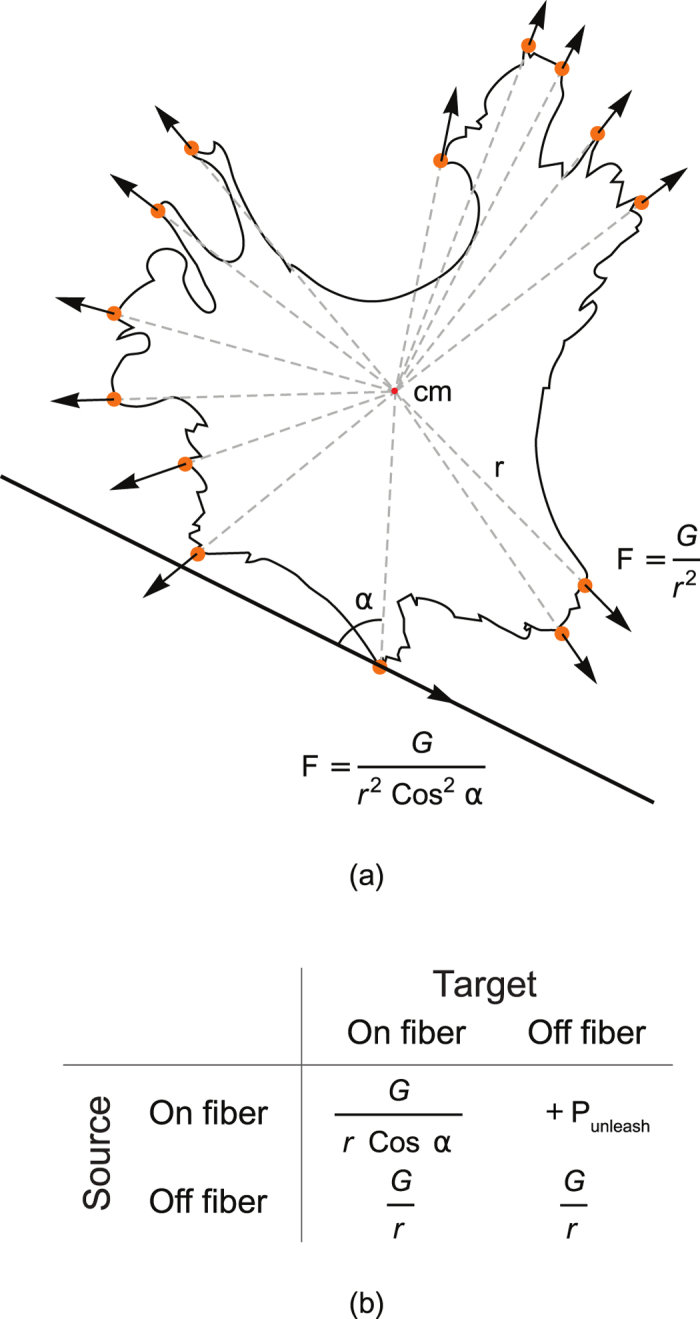
Schematic representation of cell spreading and cell–nanofibre interaction. (**a**) The cell shape was taken from the experimental database. All protrusions are marked with orange circles. Arrows show the direction of the resulting force driving the cell expansion at the attachment sites. If no guiding fibres exist, the cell spreads equidirectionally, and the forces are directed from the centre of mass of the cell (cm). If a nanofibre exists (at the bottom), and the cell is already attached to it, then the force is aligned with the fibre. (**b**) Energy terms, applied for different copy attemts of the attachment site to the non-attachment subcell, from the isotropic sibstrate to the fiber or in opposite direction.

For the cell–fibre interaction, the term (3) was substituted with (4). This term takes into account the fact that the cell increases the applied force to the substrate only if there is a reaction force from the scaffold^36^. In the case of a nanofibre, this force is always aligned with it. In obtaining the corresponding energy term, the force $G/(r\,\cos \,\alpha {)}^{2}$ was integrated with the displacement along the fibre $dr\,{\rm{c}}{\rm{o}}{\rm{s}}(\alpha )$ (see Fig. 7(a)), resulting in energy term $G/(r\,\cos \,\alpha )$, which is responsible for the direction dependence. Here, *α* is an angle between the fibre orientation (${\varphi }_{{f}_{i}}$) and the direction from ${{\rm{cm}}}_{{\sigma }_{i}}$ to the current subcell *i*. The last term in (4) is a penalty applied for copying an adhesion site from the fibre to the non-fiber region. Copying from the non-fiber to the fibre subcell is performed without energy change (see Fig. 7(b)). If the Target subcell is occupied with another attachment site–the *P*_detact_ is additionally applied, since the target attachment was destroyed. The justification for this term is given in Section I B.

Apart from the energy terms described with Equation (5), the cell shapes were also affected by two rules in the algorithm. Firstly, the copy attempts that break the connectivity of the cell were not considered and automatically rejected. Secondly, the protrusion was limited by a certain maximal length, which is specified in Table 2. Finally, if, as a result of a copy attempt, a cell disappears, then this action is also forbidden.

Finally, a penalty was added to protect the nuclei of the cell. The nuclei were assumed to be much stiffer than the cell body. Here, *P*_N_ was set to 2.0 × *P*_detach_ for all the cells that are closer to cm than to 7 *μm*.

#### Initial conditions

The cells were seeded with the density specified in Table 2 for each case. The sample was divided into ${N}_{x}\times {N}_{y}$ areas, and within these areas, the square seed of the size $\sqrt{{V}_{t}\mathrm{/10}}$ was placed in a random place within this area. There are no focal adhesions at the beginning, but during the first successful expansions of the cell border, focal adhesions are placed in newly added subcells. Their number is limited by *N*_protr_ for each cell.

#### Parameter adjustment

The parameters of the model were varied with the use of Monte Carlo algorithm to fit the experimental characteristics of the cell shapes. The best fit for the five selected parameters (area, convex coverage, elongation, number of convexes and protrusions) was found, and the corresponding parameters of the model are listed in Table 2.

#### Numerical implementation

Our GGH model was implemented on the basis of the code published as a part of ref. 61 (Protocol S1). The code for adhesive and elastic energies was unchanged, but the function for protrusion energy was added.

The space step was 2.5 *μm*, and the mesh size was 320 × 320 pixels or 0.8 mm × 0.8 mm for monolayer simulations, or 400 × 400 pixels or 1.0 mm × 1.0 mm for isolated cells (see Table 2). For electrophysiological studies, larger meshes (with the same 2.5 *μm* resolution) were generated.

The code was optimised to compute large meshes. Connectivity check was performed previously in a whole sample, and the program took *O*(*n* ^2^) time, where *n* is the number of pixels in the sample. Each cell was surrounded with a box and the connectivity was checked only within the box. Therefore, the connectivity check itself took *O*(*m*) instead of *O*(*n*), where *m* is the size of the box. The size of an average box is comparable to the size of a cell and does not change with a change in the sample size. Therefore, the whole GGH model now scales linearly (*O*(*n*)) with the sample size.

With this optimisation, it takes 3.5 hours on an Intel Core i7-3930K CPU to compute a 1 cm × 1 cm monolayer on fibres.

### Electrophysiological model

We used a monodomain ionic model of neonatal rat CM, developed by R. Majumder in 2016^39^, which is a modification of the Kohronen model with simplified calcium dynamics. The transmembrane potential in this model was determined as follows:

6 $$\frac{\partial V}{\partial t}=\nabla (D\nabla V)-\frac{{I}_{{\rm{ion}}}+{I}_{{\rm{stim}}}}{{C}_{m}},$$

where *V* is the transmembrane potential, *C*_*m*_ is the membrane capacitance, and *D* is the coupling coefficient. Coefficient *D* depends on the space variable and differs substantially for cell–cell connections or propagation within a biological cell. Each subcell in the GGH model was used as a pixel for electrophysiological simulations.

The *I*_*ion*_ is given by the sum of the following ionic currents:

7 $${I}_{ion}={I}_{Na}+{I}_{K1}+{I}_{to}+{I}_{Kr}+{I}_{Ks}+{I}_{CaL}+{I}_{NaCa}+{I}_{NaK}+{I}_{pCa}+{I}_{pK}+{I}_{bCa}+{I}_{bNa}.$$

The equations for these currents are listed in R. Majumder paper^39^.

We implemented a non-uniform distribution of gap junctions (GJs) along the cell membrane, that was previously shown in experiments^62, 63^. We assumed that more connexins were transported to the attachment sites because the directions of the actin filaments and microtubules of the cell are correlated with one another^64^. Microtubules are pathways for the transportation of proteins making ionic channels from the centriole to the membrane^65^. Actin filaments, on the contrary, polymerise from the attachment site to the cell body. Therefore, we expect more proteins to be transported to the protrusion sites, leading to a non-uniform channel distribution.

**Figure 8:**
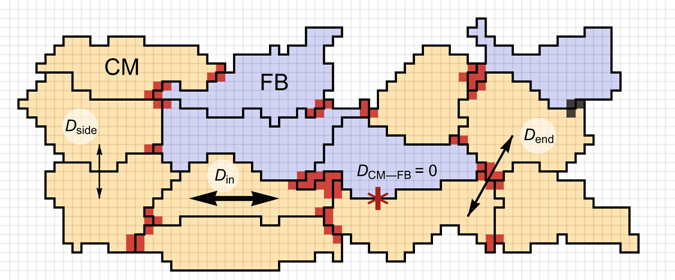
Algorithm for the coupling coefficient assignment. CMs are coloured in yellow, whereas FBs are coloured in blue. Dark red areas represent end–end type connections where the attachment sites in a GGH model are located. Between the red subcells of two different cells, the ${D}_{{\rm{end}}}$ coefficient was used. For all other connections between cells, the coupling coefficient was ${D}_{{\rm{side}}}$. For connections within a cell, the coupling coefficient was ${D}_{{\rm{in}}}$, and ${D}_{{\rm{in}}}\gg {D}_{{\rm{end}}} > {D}_{{\rm{side}}}$. No coupling occurred between CM and FB in our model.

As a result, stronger cell–cell connections were created if the protrusion of one cell touches the protrusion of the other cell. In this case, we used *D*_end-end_ (see Fig. 8). For all other connections, coefficient *D*_side-side_ was applied. This approach allowed us to obtain the functional anisotropy for a geometrically elongated cell without setting any preferred direction in the model. The coupling coefficients between CMs and FBs were equal to zero in this study.

The exact value of *D*_end-end_ was adjusted to fit the longitudinal velocity. In Fig. 6, *D*_side-side_ = 0, *D*_end-end_ = 0.01*D*_in_ in isotropic case, and 0.02*D*_in_ in anisotropic sample. In simulations with *D*_side-side_ = *D*_end-end_ = *D*_in_ the lowest possible anisotropy ratio was achieved.

#### GJ distribution

Two types of connections are considered in our model: end-to-end and side-to-side connections. The end-to-end connection was established at the sites adjoined the attachment sites. We hypothesised that more GJs are distributed along the cytoskeleton. Therefore, we proposed one more GGH-like model for channel distribution. This model has two types of cells: more-GJ and less-GJ subcells. The Hamiltonian of the model comprises only two terms:

8 $$H={H}_{{\rm{adhesive}}}+{H}_{{\rm{protr}}}=\sum _{\mathop{{\rm{neighbours}}}\limits^{(\vec{i},\vec{j})}}{J}_{{\tilde{\sigma }}_{i},{\tilde{\sigma }}_{j}}^{{\sigma }_{i},{\sigma }_{j}}+\sum _{\mathop{\text{more}-\text{GJ}}\limits^{}}\frac{G}{\rho (\vec{i},{\mathop{{\rm{cm}}}\limits^{\longrightarrow}}_{{\tilde{\sigma }}_{i}})}$$

where *G* = 25.0 is the spreading constant, and *J* is equal to 0 for the end-to-end connections between two different cells, *J*_*H*_ = 2.0 for side-to-side connections and *J*_*B*_ = 10.0 for the interactions of the contacts within one biological cell. $\tilde{\sigma }$ is the index specifying the connection type (0: less GJ, 1: more GJ), and $\sigma$ is the cell index, which was taken from the cell morphology simulation and remained constant during the channel distribution computations. This model induces the GJs to spread further from the cm (the higher is the *G*), preferably along the border (the higher is the *J*_*B*_) and preferably keeping the connection with more-GJ subcells of the other cells (*J*_*H*_). These parameters were adjusted to create a reasonable surrounding of the attachment sites. However, this model has no data for validation yet.

Finally, with the use of the resulting channel distribution from this simulation, coupling coefficients were assigned. The end-to-end coupling coefficient *D*_end-end_ was applied if, on both sides of the border, a subcell of a more-GJ type exists. For all other intercellular connections, *D*_side-side_ was used.

The conduction velocity highly depends on the five parameters (*G*_*N*_, *J*_*H*_, *J*_*B*_, *D*_end-end_/*D*_in_, *D*_side-side_/*D*_in_). If *D*_side-side_ ≪ *D*_end-end_, the anisotropy mainly depends on channel distribution, and *D*_end-end_ regulates the longitudinal velocity. However, in our irregular discrete model, the velocity ratio was limited by the geometrical anisotropy. From this, we concluded that morphological studies provide essential constraints on the excitation propagation. The details of cell-to-cell signal transduction should be studied further.

#### Numerical implementation

We used graphic processing units (GPUs) for the detailed ionic model integration. GPUs are very efficient for excitable media simulations with large sample sizes. The ionic model was already implemented in CUDA in our group^39, 66^, but the coupling term of the model was significantly changed in the current study.

The equation (6) was discretised and solved with the alternating direction (AD) implicit method. The hidden *n* + 1/2 time step was added to implement it. The currents were computed with the parameters from the previous time step *n*. The diffusion was first computed explicitly in one direction (taking time step *n*) and implicitly in the other direction during the 1/2 time step:

9 $$\begin{array}{ccc}\frac{{V}^{n+1/2}-{V}^{n}}{\tau /2} & = & \frac{{D}_{{\rm{l}}{\rm{e}}{\rm{f}}{\rm{t}}}({V}_{{\rm{l}}{\rm{e}}{\rm{f}}{\rm{t}}}^{n+1/2}-{V}^{n+1/2})+{D}_{{\rm{r}}{\rm{i}}{\rm{g}}{\rm{h}}{\rm{t}}}({V}_{{\rm{r}}{\rm{i}}{\rm{g}}{\rm{h}}{\rm{t}}}^{n+1/2}-{V}^{n+1/2})}{{h}^{2}}+\\  &  & +\frac{{D}_{{\rm{t}}{\rm{o}}{\rm{p}}}({V}_{{\rm{t}}{\rm{o}}{\rm{p}}}^{n}-{V}^{n})+{D}_{{\rm{b}}{\rm{o}}{\rm{t}}{\rm{t}}{\rm{o}}{\rm{m}}}({V}_{{\rm{b}}{\rm{o}}{\rm{t}}{\rm{t}}{\rm{o}}{\rm{m}}}^{n}-{V}^{n})}{{h}^{2}}+{I}_{{\rm{i}}{\rm{o}}{\rm{n}}}^{n}\end{array}$$

Afterwards, the directions were swapped, and the procedure was repeated. On each half-step and at each point, there are three potentials *V* taken at a future *n* + 1/2 time step. To find them, let us rewrite the equation (9) in the form $A{V}^{n+\mathrm{1/2}}={b}^{n}$, where *A* is a constant matrix. This matrix is tridiagonal. Therefore, the tridiagonal solver from the cuSPARSE library was used to find *V* ^*n+*1/2^ from *V* ^*n*^. *V* ^*n+*1^ was found similarly from *V* ^*n+*1/2^ after direction exchange.

The reason why we use the AD method is that the space step in our model should be *h* = 2.5 *μm*, the same as that in the GGH model. The explicit algorithm is limited by the Courant number, which is equal to *Dh*/*τ* ^2^ and should not exceed 1/2 for differential equations of parabolic type. Normally, in our computations for arrhythmia studies, *h* is equal to 250 *μm* ^66^, which is comparable to the cell size, and the time step is 20 *μs*. If the space step is reduced to 2.5 *μm*, then the time step in the explicit model becomes 2 *ns*, and the computational time increases 10^8^ times. Therefore, the explicit method is not applicable to subcellular studies.

On the other hand, implicit or semi-implicit methods possess absolute stability, and larger time steps could be used. The approximation of the solution will be affected on the subcellular level, but because the important spatial harmonics of the solution are much bigger than the cell or subcell size, then the macroscopic solution of the wavefront propagation will be calculated correctly. We took *h* = 2.5 *μm* and the time step *t* = 1 *ms*; the highest coupling coefficient was *D* = 1 cm^2^/s.

The anisotropy was tested in the 4.96 mm × 4.96 mm samples (1984 × 1984 grid points). It was cut from a larger 5.0 mm × 5.0 mm mesh from the GGH model to reduce the margin size without the cells and to fit the GPU architecture better. The program runs on GPU most efficiently if the size of the sample is divisible by the block size (32 × 32 in our simulations). For the simulation of the 40 ms activity, it took 75 minutes on the GPU. For larger samples, the computation time scales as O(*n* ^*2*^), where *n* is the total number of grid points.

### Code availability

The custom computer code is available from the corresponding authors upon reasonable request. The file with energy terms can be found in Supplementary Materials Dataset 5.

## Electronic supplementary material

Dataset 1

Video 3A

Video 3B

Video 3C

Video 3D

Video 3E

Video 3F

Video 4A

Video 4B

Video 4C

Video 4D

Supplementary Information
