# Supplementary figures and images for: Virtual cardiac monolayers for electrical wave propagation
Source: Sci Rep. 2017 Aug 11;7:7887. doi: 10.1038/s41598-017-07653-3 (PMC5554264; doi:10.1038/s41598-017-07653-3)

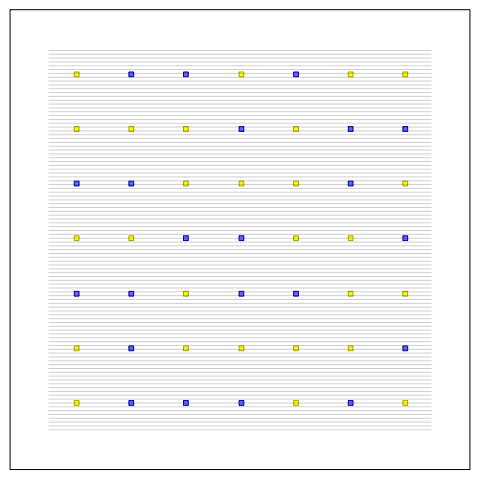

Supplement: Supplementary file 2 — Video 3A [file 41598_2017_7653_MOESM2_ESM.gif]

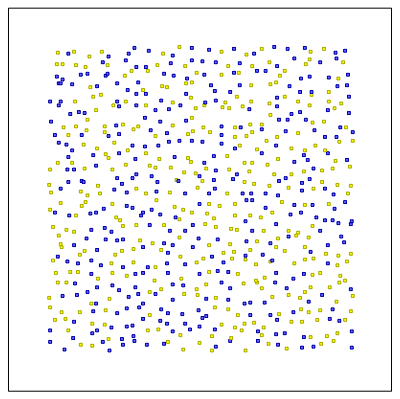

Supplement: Supplementary file 3 — Video 3B [file 41598_2017_7653_MOESM3_ESM.gif]

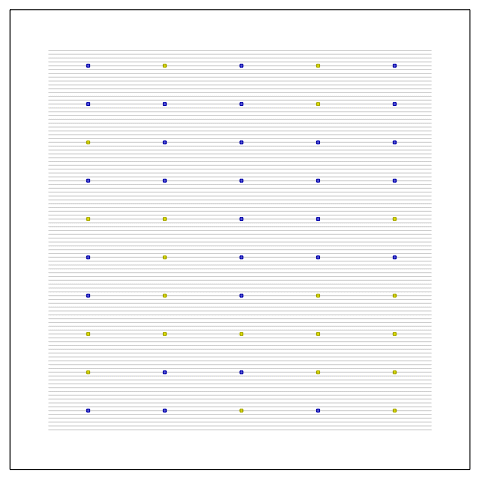

Supplement: Supplementary file 4 — Video 3C [file 41598_2017_7653_MOESM4_ESM.gif]

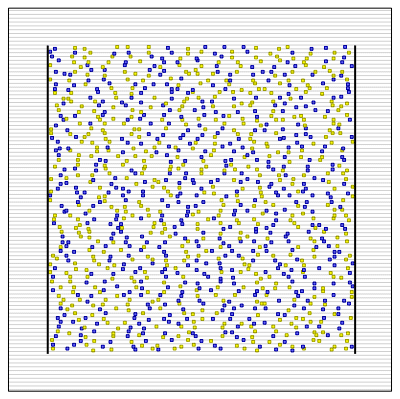

Supplement: Supplementary file 5 — Video 3D [file 41598_2017_7653_MOESM5_ESM.gif]

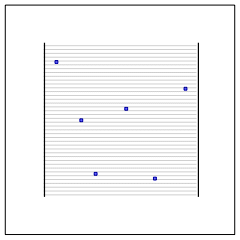

Supplement: Supplementary file 6 — Video 3E [file 41598_2017_7653_MOESM6_ESM.gif]

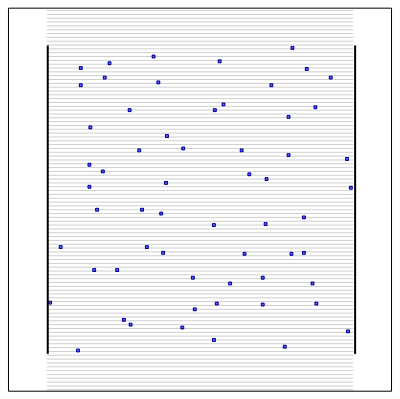

Supplement: Supplementary file 7 — Video 3F [file 41598_2017_7653_MOESM7_ESM.gif]
