# Supplementary material for: Virtual cardiac monolayers for electrical wave propagation
Source: Sci Rep. 2017 Aug 11;7:7887. doi: 10.1038/s41598-017-07653-3 (PMC5554264; doi:10.1038/s41598-017-07653-3)
Supplement: Supplementary file 12 — Supplementary Information [file 41598_2017_7653_MOESM12_ESM.pdf]

Virtual cardiac monolayers for electrical wave propagation

Nina Kudryashova,[1,2] Valeriya Tsvelaya,[2] Konstantin Agladze,[2,*] and Alexander Panfilov[1,2,*]

[1] *Department of Physics and Astronomy, Gent University*

[2] *Laboratory of Biophysics of Excitable Systems, Moscow Institute of Physics and Technology*

SUPPLEMENTARY MATERIALS

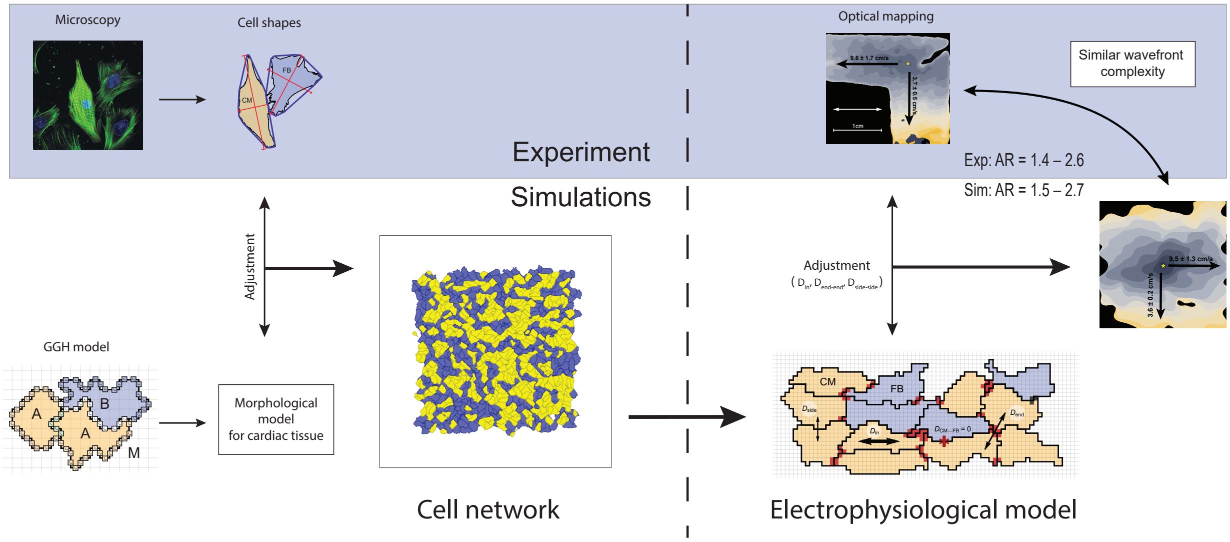

Figure S1. The scheme of the current joint in silico and in vitro study. The experimental measurements are shown on the blue background. First, the GGH model was extended to describe cardiac tissue. In parallel, cell shapes data was collected and analysed in experiment. The model was then validated with the use of these data. As a result, the virtual model for cardiac tissue was developed. In the second part of our study we aimed to reproduce propagation of the electrical waves. The virtual cells generated in the first (left) part of our study were used. These cells comprise of a number of the subcell of the regular grid. Different coupling coefficients were assigned to the subcell-to-subcell connections in this grid. These coefficients describe wave propagation along the cell and between the cells. End-to-end electrical connections are considered to be stronger due to the higher concentrations of the GJs in the end part of the cell. The resulting electrophysiological model was validated with the optical mapping experiments. The wave pattern for a particular sample was reproduced with adjustment of D_{end} and D_{side}. Varying D_{side}/D_{end}, the range of the electrical anisotropy ratios in our model was found. This range fits the range of anisotropies, observed in experiments. Therefore, this image represents the overview on our study and highlights the most important results.

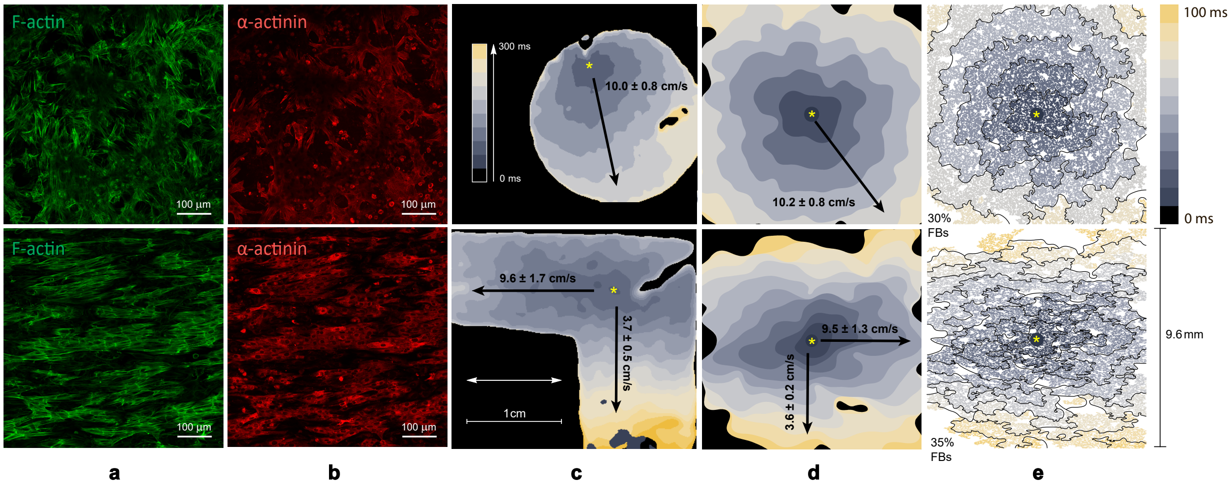

Figure S2. Experimental results acquired with the optical mapping of isotropic and anisotropic samples compared to simulated ones (extended version of Figure 6).

(a) F-actin staining; Upper image shows the isotropic sample, whereas the lower image shows the anisotropic sample on nanofibers.

(b) α-actinin staining, specific for CMs.

(c) Activation maps for the corresponding samples. The yellow star shows the place where stimulation with an electrode was applied. The white arrow shows the preferred direction of the fibres in the sample. Activation time is colour coded (0 — 300 ms).

(d) Corresponding simulations of the wave propagation in samples with 30% and 35% FBs in isotropic and anisotropic samples, respectively. The same filters with the same kernel size were applied to the simulated data, as for the experimental data in column (c).

(e) Detailed representation of the corresponding computer simulation before the filters were applied.
